# Diffuse alveolar hemorrhage during alemtuzumab infusion in a patient with multiple sclerosis: a case report

**DOI:** 10.1186/s40360-018-0267-5

**Published:** 2018-11-19

**Authors:** Aija Zuleron Myro, Gisle Bjerke, Svetozar Zarnovicky, Trygve Holmøy

**Affiliations:** 1 Department of Neurology, Akershus University Hospital, Postboks 1000, 1478 Lørenskog, Norway; 2 Department of Pulmonary Medicine, Akershus University Hospital, Lørenskog, Norway; 3 Department of Radiology, Akershus University Hospital, Lørenskog, Norway; 4 Institute of Clinical Medicine, University of Oslo, Oslo, Norway

**Keywords:** Multiple sclerosis- alemtuzumab-adverse events-diffuse alveolar hemorrhage-case report

## Abstract

**Background:**

Diffuse alveolar bleeding is a potentially life-threatening condition that can be induced by several drugs. Whereas fatal cases have been reported in patients treated for other indications, no report have so far been published in a patient with multiple sclerosis treated with alemtuzumab.

**Case presentation:**

We report a case of alemtuzumab-induced diffuse alveolar bleeding in a 29 year old woman with relapsing remitting multiple sclerosis. The patient developed acute shortness of breath, chest pain on inspiration and haemoptysis following the second infusion of alemtuzumab during the first treatment cycle. Computed tomography showed bilateral alveolar opacities. Bronchoscopy and broncho-alveolar lavage showed persistently bloody return with no evidence of infection. The symptoms resolved completely without treatment and control computed tomography performed one week later showed total resolution of pulmonary infiltrates.

**Conclusion:**

This is the first published report of diffuse alveolar bleeding in a patient with multiple sclerosis treated with alemtuzumab. Four similar cases in patients treated for multiple sclerosis and several fatal cases in patients treated for other conditions are registered at the World Health Organization database of suspected adverse events (VIgiBase©), underscoring that this is a serious and possibly under-recognized complication of alemtuzumab which can also occur in the treatment of multiple sclerosis. The clinician should consider the possibility of diffuse pulmonary haemorrhage in patients with sudden onset of respiratory distress and haemoptysis following administration of alemtuzumab for multiple sclerosis.

## Background

Alemtuzumab is a monoclonal antibody used in the treatment of relapsing remitting multiple sclerosis (MS), targeting CD-52 which is expressed on mature lymphocytes and to a lesser extent on myeloid cells [1]. Infusion reactions occur in > 90% of patients receiving alemtuzumab, and are serious in 3% [2]. We report a patient who developed diffuse alveolar bleeding, a potentially life-threatening condition not previously published in an MS patient treated with alemtuzumab.

## Case presentation

A 29 year old woman with a previous history of migraine, mild asthma, congenital asymptomatic bicuspid aorta valve accidentally discovered during routine examination, missed abortion and anembryonic pregnancy was diagnosed with relapsing-remitting MS. She was treated with interferon beta1-b for five years, until it was decided to escalate the treatment due to new gadolinium enhancing MRI lesions and a sensory attack. The Expanded Disability Status Scale score was 2,0. Tests for tuberculosis, HIV, hepatitis B and C, routine blood and urine analyses, as well as respiratory examination and chest X-ray were negative. She had stopped smoking four years previously, and stopped using interferon beta 1b four months prior to the first alemtuzumab infusion because she wished to get pregnant.

The patient received standard premedication with 1000 mg methylprednisolone, 10 mg cetirizine, 1000 mg paracetamol and 400 mg acyclovir per day before each alemtuzumab infusion (12 mg per day). Prior to administration of alemtuzumab hypotension (70/35 mmHg) and bradycardia (45 beats per minute) was noticed and patient reported mild dizziness that improved after administration of Ringer’s acetate. A vasovagal reaction was suspected. For this reason, the alemtuzumab infusion was started at a low rate (12 ml/hour). Except mild headache that was treated with paracetamol and ibuprofen no infusion-associated reactions were observed the first day. Blood pressure and heart rate were normal during the alemtuzumab infusion.

At the end of the second alemtuzumab infusion, 24 h after the start of the first infusion, the patient developed chest pain on inspiration, shortness of breath, and cough. Four hours later she started coughing up bright red blood tinged sputum without clots. Body temperature, blood pressure, heart sounds and oxygen saturation were normal. Electrocardiogram showed sinus bradycardia at 48 beats/min. Auscultation revealed crepitations over the right lung, and chest x-ray showed corresponding shadowing. Platelet and leukocyte counts two hours after symptom onset were normal, and c-reactive protein was 26 mg/l (ref. < 5). Arterial blood gas analysis four hours after onset of haemoptysis was normal except for pO2 at the lower reference limit (11,0 kPa; ref. 11,0–14,4) and elevated lactate 1,3 mmol/l (ref.0,4-0,8). Haemoglobin fell from 12,0 g/dl (ref. 3, 7–15 g/dl) before the first infusion to 10,5 g/dl the day after onset of haemoptysis. Urinary analysis and serum creatinine remained normal.

**Fig. 1:**
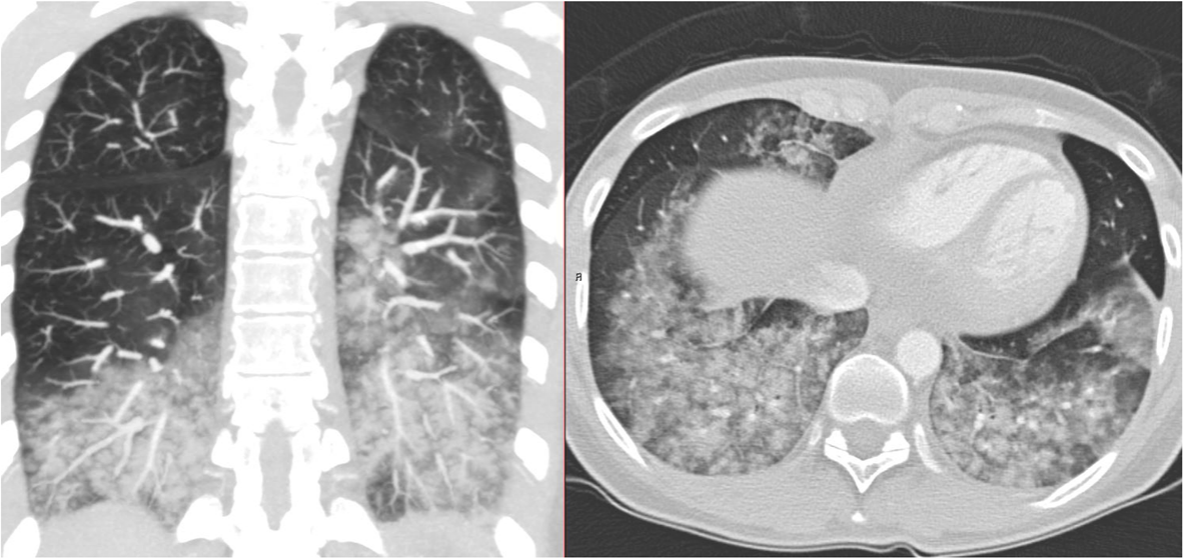
Computed tomography of the chest taken 11 h after onset of hemoptysis showing bilateral alveolar opacities

Computed tomography (CT) pulmonary angiography performed shortly after onset of haemoptysis showed extensive bilateral upper and lower lobe opacities with centrilobular distribution and minimal (up to 5 mm) bilateral pleural effusions without evidence of pulmonary embolism (Fig. 1). Interlobular septal thickening and dependent gradient were not present. The heart size was normal. At bronchoscopy performed 60 h after onset of haemoptysis the bronchoalveolar lavage (BAL) fluid was persistently macroscopically markedly bloody, without dilution on successive aliquots. There were no evidence of pathogenic bacteria, viruses, or atypical cells. Differential cell count of the BAL revealed 6% macrophages without hemosiderin inclusions on iron staining and 94% neutrophils indicating acute lung injury. Microscopy of BAL fluid showed many erythrocytes but counting in successive aliquots was not performed.

The patient remained stable from a respiratory point of view with normal vital parameters. No treatment for lung haemorrhage was given. Chest pain and haemoptysis resolved in two days. At discharge from hospital one week after onset of haemoptysis CT showed total resolution of pulmonary opacities and pleural effusions on the right side, and unchanged minimal amounts of pleural effusion on the left side. Her cough resolved after four weeks, and she has thereafter not experienced respiratory symptoms.

Alemtuzumab was discontinued after the second infusion. Her MS has remained clinically and radiologically stable, and new treatment has not been initiated because of prolonged lymphopenia (0,33 10*9/l 13 months after alemtuzumab infusions).

## Discussion and conclusions

Diffuse alveolar haemorrhage (DAH) is a clinical-pathological syndrome with accumulation of red blood cells in the alveolar spaces due to injury of the pulmonary microcirculation, that can be caused by immune and coagulation disorders, heart disease, infections and drugs [3]. The mechanism of drug-induced DAH is heterogeneous and involves pulmonary capillaritis, direct toxic damage of the alveolar epithelium and basement membrane and coagulation defects [4]. The typical symptoms are acute or subacute haemoptysis, cough and dyspnea. Haemoptysis can be absent in 30% of patients [3].

Macroscopically haemorrhagic BAL fluid is considered diagnostic of acute alveolar haemorrhage [3]. CT typically shows ground-glass opacities and consolidation reflecting alveolar filling, which may also be seen in pulmonary oedema [3]. Since our patient had normal heart size, centrilobular distribution of opacities, there was no evidence of interlobular septal thickening and dependent gradient usually seen in lung oedema and only minimal pleural effusion, we find DAH due to pulmonary oedema less likely.

Pleural effusion is uncommon in DAH [3] but can be seen in pleuritis that has been reported as a serious infusion reaction in an MS patient treated with alemtuzumab [5]. Pericardial effusion along with pneumonitis was also described in another MS patient after the second alemtuzumab infusion [6]. As our patient also had pain on inspiration, we consider pleuritis caused by alemtuzumab as the most likely cause of the minimal pleural effusions.

Haemosiderin-laden macrophages are typically present in the BAL fluid 36–72 h after diffuse alveolar bleeding [3], but were not seen in our patient 60 h after symptom onset. It should be noted that the target antigen of alemtuzumab (CD52) is also expressed on myeloid cells including macrophages [7], and that the function of such cells is severely impaired in the early phase after alemtuzumab infusion [8]. It is therefore conceivable that the uptake of haemosiderin by macrophages could be perturbed shortly after alemtuzumab infusion. The two previously published case reports of alemtuzumab-associated alveolar haemorrhage do not mention whether this finding was present [9, 10].

Alveolar haemorrhage can be induced by several drugs including monoclonal antibodies [4, 11]. Two reports on patients with Alport’s syndrome who received alemtuzumab as a part of immunosuppressive induction protocols during kidney transplantation have been published [9, 10]. Like our patient both these patients received premedication with steroids before administration of alemtuzumab, and in one BAL fluid was also positive for polymorphs [10]. In contrast to our patient who received 12 mg alemtuzumab on two consecutive days, both these patients received 30 mg alemtuzumab as one single subcutaneous or intravenous injection at the transplantation day, and both developed acute respiratory failure and haemoptysis the second day after alemtuzumab administration. Unlike our patient who recovered without any treatment after cessation of alemtuzumab, both developed severe respiratory failure and required mechanical ventilation, and were treated with steroids, plasma exchange and dialysis due to acute tubular necrosis. One of these patients died due to worsening respiratory function and failing graft function [10].

Alport’s syndrome possibly predisposes to alveolar haemorrhage due to altered collagen structure [10]. Our patient has an asymptomatic bicuspid aortic valve which can be associated with connective tissue disorders [12]. High throughput sequencing of 44 genes associated with connective tissue disorders did however not reveal relevant pathology.

In total, 22 cases of alveolar haemorrhage associated with alemtuzumab have been reported to VigiBase©, the World Health Organization’s international database for suspected adverse drug reactions [13]. Including our patient, five of these received alemtuzumab for MS. Like our patient all other patients treated for MS received two infusions, and all recovered. Unfortunately, no further details about treatment are available at VigiBase©. The remaining 17 patients received alemtuzumab as part of immunosuppression for transplantation or for haematological malignancies, and 14 of these died [13].

The mechanism underlying alveolar bleeding induced by alemtuzumab is unknown. Alemtuzumab is associated with secondary autoimmune diseases, including nephritis with antibodies against basal membranes that could induce alveolar haemorrhage [2]. Anti-glomerulus basement membrane antibodies and anti-neutrophil cytoplasmic antibodies were however negative, and the short interval from treatment to symptoms also excludes this possibility. Cytokine-release syndrome is the most common cause of infusion reactions associated with alemtuzumab, and may cause a number of symptoms including headache, rash, pyrexia, nausea, dyspnea, chest discomfort and hypotension [2, 14]. Cytokine-release syndrome associated with monoclonal antibodies is however usually a first dose phenomenon with decreasing severity during subsequent administrations [14, 15]. Our patient had only mild headache and no other evidence of cytokine-release syndrome during the first alemtuzumab infusion. Sinus bradycardia is a common side effect of alemtuzumab [16], and was not likely associated with the alveolar bleeding.

Notably, alemtuzumab activates both cellular and complement dependent cytotoxicity and induces profound and immediate effects on the innate immune system [8]. It can be speculated that effector mechanisms induce acute inflammation that in some cases may damage membranes and cells not expressing the CD52 target molecule.

The treatment of DAH associated with drugs is cessation of the suspected medication, and in more severe cases high dose steroids [4]. Plasma exchange is used in selected autoimmune disorders [3] and can be considered in severe cases of drug-induced DAH but its effectiveness is questionable [4].

This case along with those previously reported to VigiBase© [13], as well as published cases treated for other diseases [9, 10], underscore that diffuse pulmonary haemorrhage is a potentially fatal complication of alemtuzumab, usually occurring after few infusions. Clinicians should consider this diagnosis in patients who develop sudden onset of respiratory distress and haemoptysis during or shortly after administration of alemtuzumab.

## Abbreviations

BAL: Bronchoalveolar lavage
CT: Computed tomography
DAH: Diffuse alveolar haemorrhage
MS: Multiple sclerosis
